# Complete Genome Sequence of *Plesiomonas shigelloides* Type Strain NCTC10360

**DOI:** 10.1128/genomeA.01031-16

**Published:** 2016-09-22

**Authors:** Sarah Alexander, Mohammed-Abbas Fazal, Edward Burnett, Ana Deheer-Graham, Karen Oliver, Nancy Holroyd, Julian Parkhill, Julie E. Russell

**Affiliations:** a Culture Collections, Public Health England, London, United Kingdom; b Wellcome Trust Sanger Institute, Wellcome Trust Genome Campus, Hinxton, United Kingdom

## Abstract

*Plesiomonas shigelloides* is a Gram-negative rod within the *Enterobacteriaceae* family. It is a gastrointestinal pathogen of increasing notoriety, often associated with diarrheal disease. *P. shigelloides* is waterborne, and infection is often linked to the consumption of seafood. Here, we describe the first complete genome for *P. shigelloides* type strain NCTC10360.

## GENOME ANNOUNCEMENT

*Plesiomonas shigelloides* is a flagellated, facultative anaerobic, oxidase-positive, and rod-shaped bacterium which has recently been classified within the *Enterobacteriaceae* family. *P. shigelloides* is the only species in the *Plesiomonas* genus, and this species historically has been classified in the *Vibrionaceae* family and the *Aeromonas* genus (1, 2). To date, this bacterium has been associated mainly with aquatic environments and organisms that reside in aquatic environments. *P. shigelloides* has been isolated from streams, lakes, estuarine waters, mammals, crustaceans, mollusks, reptiles, amphibians, and fish (3).

*P. shigelloides* is a known human pathogen of increasing significance. Principally, it has been linked to gastrointestinal infection, mainly diarrheal diseases, and also in rarer isolated cases to invasive infections, including sepsis, meningitis, and pseudoappendicitis (4). It is possible that cases of *P. shigelloides* are underreported, because although this bacterial species can be identified using a range of commercially available biochemical test systems (Vitex 2 system and Phoenix 100 ID/AST NID card), its low abundance in fecal samples and small colony size can result in its presence being overlooked (4). To date, there is only one other draft genome sequence available for *P. shigelloides*, that from strain 302-73. This sequence, however, is composed of 13 scaffolds containing 389 contigs (5). Here, we report the first available complete genome sequence of *P. shigelloides* from the type strain NCTC10360.

Genomic DNA was extracted from 24-h pure culture using the MasterPure DNA extraction kit (Epicentre, WI, USA). Whole-genome sequencing (WGS) was performed using the Pacific Bioscience (PacBio) RSII platform. DNA was sheared to 15 kb, and 10 kb- to 20 kb-library and sequencing were performed utilizing C4/P6 chemistry. Genome assembly was performed using an automated assembly pipeline (HGAP/Quiver software package), and annotation was performed with Prokka.

The genome of strain NCTC10360 was 3,405,979 bp, which was confined to a single chromosome; no plasmids were identified in this strain. The average G+C content of the sequence was 52%. In total, there were 3,093 coding sequences, including 11 rRNA operons and 113 tRNA genes.

### Accession number(s).

The complete genome sequence has been deposited to the European Nucleotide Archive under accession number LT575468.

## References

[1] Schubert RH 1974 Genus III *Plesiomonas*, p 348–349. *In* Bergey’s manual of determinative bacteriology, 8th ed. Williams & Wilkins, Baltimore, MD.

[2] Ruimy R, Breittmayer V, Elbaze P, Lafay B, Boussemart O, Gauthier M, Christen R 1994 Phylogenetic analysis and assessment of the genera *Vibrio*, *Photobacterium*, *Aeromonas*, and *Plesiomonas* deduced from small-subunit rRNA sequences. Int J Syst Bacteriol 44:416–426. 7520733 10.1099/00207713-44-3-416

[3] Jagger TD 2000 *Plesiomonas shigelloides*: a veterinary perspective. Infect Dis Rev 2:199.

[4] Janda JM, Abbott SL, McIver CJ 2016 *Plesiomonas shigelloides* revisited. Clin Microbiol Rev 29:349–374. doi:10.1128/CMR.00103-15. 26960939 PMC4786884

[5] Piqué N, Aquilini E, Alioto T, Miñana-Galbis D, Tomás JM 2013 Genome sequence of *Plesiomonas shigelloides* strain 302-73 (serotype O1). Genome Announc 1(4):e00404-13. doi:10.1128/genomeA.00404-13. 23814109 PMC3695437

